# Special Issue “Efficiency in Kinesiology: Innovative Approaches in Enhancing Motor Skills for Athletic Performance, 3rd Edition”

**DOI:** 10.3390/jfmk10030303

**Published:** 2025-08-05

**Authors:** Vincenzo Sorgente, Diego Minciacchi

**Affiliations:** Kinesiology and Motor Control (Ki.Mo.Co.) Laboratory, Department of Experimental and Clinical Medicine, Physiological Sciences Section, University of Florence, 50134 Florence, Italy; diego.minciacchi@unifi.it

## 1. Introduction

The third edition of the Special Issue “Efficiency in Kinesiology: Innovative Approaches in Enhancing Motor Skills for Athletic Performance 3.0” presents a stimulating variety of contemporary research focusing on the multifaceted nature of motor performance optimization. This new collection contributes to the growing body of knowledge in kinesiology by highlighting diverse methodological and technological innovations applied across various healthy active and sporting contexts: https://www.mdpi.com/journal/jfmk/special_issues/95TTS9M0N6 (accessed on 10 July 2025).

Recently, substantial evidence has refined knowledge on the role of biomechanical and technical training in enhancing motor performance [1]. However, the rapid evolution of physical activity and sport science has introduced novel tools and assessment strategies, some of which have gained popularity without rigorous validation, while others, despite strong empirical support, have yet to be successfully integrated into the real-world of fitness and of athletic settings.

In light of the ongoing demand for superior performance, developing and disseminating scientifically grounded, innovative strategies to enhance motor efficiency is imperative. Such advancements are critical not only for elite athletes but also for students and recreationally active populations [2,3].

This Special Issue addresses key gaps by examining how motor skills and performance can be improved through evidence-based interventions. The ten contributions here enclosed span a wide range of sports—including basketball, hurling, badminton, pickleball, American football, table tennis, and padel—each presenting unique challenges that require tailored performance evaluation and training approaches. By showcasing multidimensional perspectives, this collection offers valuable insights into grounding motor behavior and performance.

## 2. Overview of Published Articles

Contribution 1 investigated the effectiveness of the Burpee Movement Program (BMP) in assessing and developing strength–endurance capacities. Using smartphone-based accelerometry, performance in burpees was correlated with bench press, squat output, and shuttle-run endurance. Findings support the use of BMP as a practical strength–endurance tool, with potential for diagnostic application pending further research.

Contribution 2 analyzed the relationship between physical load and game outcomes in elite under-18 basketball players. Using inertial microsensors, the study revealed that physical demands—particularly player load and steps—were stronger predictors of quarter outcomes than individual performance ratings. These results underscore the strategic importance of load monitoring during competition.

Contribution 3 examined how varying levels of visual feedback influence fine motor control during handwriting tasks. Increased visual information improved accuracy and movement smoothness, although at the cost of execution speed. The study provides key insights into sensorimotor integration and has potential implications for motor learning and rehabilitation protocols.

Contribution 4 profiled the physical demands placed on elite hurling referees across different competitions using GPS tracking. Despite minor variations across divisions and match phases, overall activity levels were consistent, highlighting the necessity of tailored conditioning programs to match referees’ unique movement patterns and intensity requirements.

Contribution 5 evaluated how elite badminton players optimize tournament participation and recovery strategies, especially during Olympic games. Top performers exhibited strategic scheduling, balancing high-level competition exposure with sufficient rest. These findings advocate for individualized planning to sustain peak performance and ranking advancement.

Contribution 6 delivered a notational analysis of women’s singles pickleball, revealing key performance patterns. Most points were won by the server and decided by unforced errors, particularly in forehand strokes. The tactical insights gained can inform skill development and coaching strategies in this rapidly growing sport.

Contribution 7 assessed the effects of vibration foam rolling treatments of varying durations on muscle skin temperature and muscle activation. A 30 s treatment was found to be most effective in reducing muscle activity while maintaining strength, suggesting a promising intervention for muscle efficiency and rehabilitation applications.

Contribution 8 profiled collegiate American football players by position group, examining variations in body composition, strength, power, and joint kinematics. While significant differences in strength and composition emerged across groups, joint mobility remained consistent—suggesting a shared need for maintaining functional kinematics in performance development.

Contribution 9 investigated jumping capacity and limb asymmetries in adolescent table tennis players. While males showed greater jump performance, females exhibited superior elasticity indices. Significant leg asymmetries were present in both sexes, providing actionable data for training and performance monitoring in youth table tennis.

Contribution 10 explored the impact of an 8-week padel training program on strength and power in untrained young health players. Improvements were observed in sprint performance and handgrip strength, suggesting that padel may serve as an effective alternative to traditional sports in enhancing youth physical fitness.

## 3. Conclusions

This Special Issue highlights the dynamic and interdisciplinary nature of contemporary research in kinesiology, particularly regarding the enhancement of motor skills and athletic performance. The featured studies offer novel perspectives and practical applications, from monitoring load in competitive settings to evaluating fine motor control and implementing sport-specific interventions in diverse populations.

Collectively, the findings underscore the importance of adopting evidence-based strategies tailored to the specific physiological, biomechanical, and cognitive demands of each motor activity and sport. Furthermore, these investigations reflect a growing consensus on the need for integrated, technology-supported approaches in performance enhancement, long-term athlete development, and motor behaviors for active populations.

As sports and exercise sciences continue to evolve, sustained collaboration between researchers, practitioners, and on-field experts is critical in translating innovation into practice—ensuring that active populations, emerging talent, and elite athletes benefit from the latest advancements in motor efficiency and training methodologies [4].

Given the success of the third edition of this Special Issue, we have launched a fourth edition, for which we hope to receive contributions to keep fine-tuning novel approaches to enhance motor skills in sports and general fitness.
